# Crystal structure of (benzene­carbo­thio­amide-κ*S*)chloridobis­(tri­phenyl­phosphane-κ*P*)silver(I)

**DOI:** 10.1107/S1600536814015992

**Published:** 2014-08-01

**Authors:** Wattana Ruangwut, Chaveng Pakawatchai

**Affiliations:** a Department of Chemistry and Center of Excellence for Innovation in Chemistry, Faculty of Science, Prince of Songkla University, Hat Yai, Songkhla 90112, Thailand

**Keywords:** crystal structure, Ag^I^ complex, benzene­carbo­thio­amide, hydrogen bonds

## Abstract

In the mononuclear mixed-ligand title complex, [AgCl(C_7_H_7_NS)(C_18_H_15_P)_2_], the Ag^I^ ion is four coordinated by one S atom of a benzene­carbo­thio­amide ligand, two P atoms of two tri­phenyl­phosphane ligands and one chloride ion, displaying a distorted tetra­hedral coordination geometry. In the crystal, pairs of N—H⋯Cl hydrogen bonds form inversion dimers. An intra­molecular N—H⋯Cl hydrogen bond is also observed.

## Related literature   

For relevant examples of structures of Ag^I^ complexes, see: Aslanidis *et al.* (1997 ▶); McFarlane *et al.* (1998 ▶); Cox *et al.* (2000 ▶); Dennehy *et al.* (2007 ▶); Nimthong *et al.* (2008 ▶). For potential applications of related complexes, see: Isab *et al.* (2010 ▶); Nawaz *et al.* (2011 ▶).
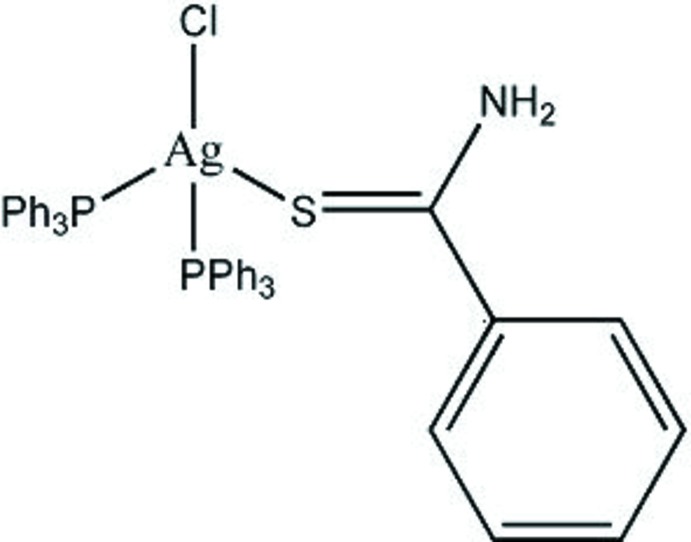


## Experimental   

### Crystal data  

[AgCl(C_7_H_7_NS)(C_18_H_15_P)_2_]
*M* *_r_* = 805.05
Monoclinic, 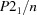

*a* = 14.2405 (7) Å
*b* = 13.9271 (7) Å
*c* = 19.0774 (10) Å
β = 98.711 (1)°
*V* = 3740.0 (3) Å^3^
*Z* = 4
Mo *K*α radiation
μ = 0.78 mm^−1^
*T* = 293 K
0.30 × 0.16 × 0.10 mm

### Data collection  

Bruker SMART APEX CCD diffractometer
Absorption correction: multi-scan (*SADABS*; Sheldrick, 2003 ▶) *T*_min_ = 0.861, *T*_max_ = 0.923
51284 measured reflections
9308 independent reflections
8544 reflections with *I* > 2σ(*I*)
*R*_int_ = 0.031

### Refinement  

*R*[*F* ^2^ > 2σ(*F* ^2^)] = 0.033
*wR*(*F* ^2^) = 0.075
*S* = 1.13
9308 reflections
442 parameters
H-atom parameters constrained
Δρ_max_ = 0.72 e Å^−3^
Δρ_min_ = −0.39 e Å^−3^

### 

Data collection: *SMART* (Bruker, 1998 ▶); cell refinement: *SAINT* (Bruker, 2003 ▶); data reduction: *SAINT*; program(s) used to solve structure: *SHELXS97* (Sheldrick, 2008 ▶); program(s) used to refine structure: *SHELXL2012* (Sheldrick, 2008 ▶), *SHELXLE* (Hübschle *et al.*, 2011 ▶); molecular graphics: *Mercury* (Macrae *et al.*, 2008 ▶); software used to prepare material for publication: *SHELXL97* and *publCIF* (Westrip, 2010 ▶).

## Supplementary Material

Crystal structure: contains datablock(s) I. DOI: 10.1107/S1600536814015992/lh5718sup1.cif

Structure factors: contains datablock(s) I. DOI: 10.1107/S1600536814015992/lh5718Isup2.hkl

**Fig. 1.**
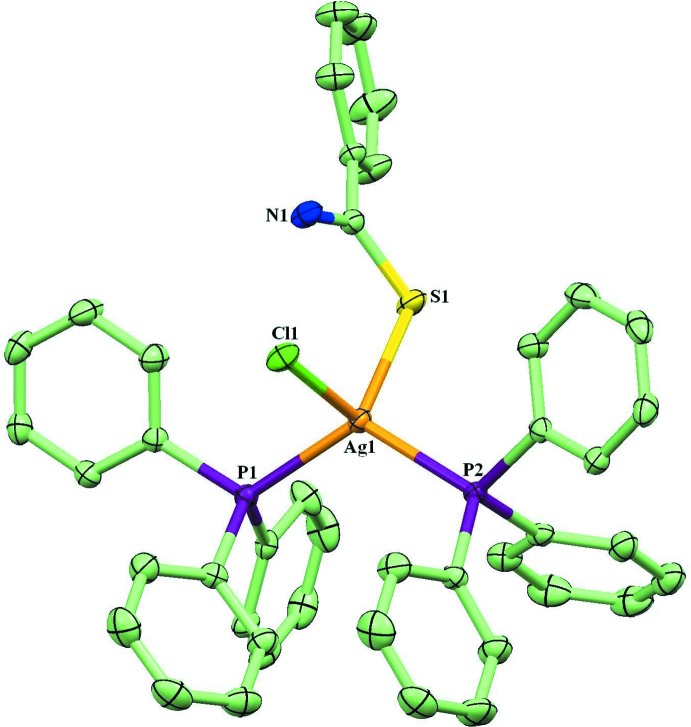
The molecular structure with displacement ellipsoids drawn at the 50% probability level and H atoms are omitted for clarity.

. DOI: 10.1107/S1600536814015992/lh5718fig1.tif
The mol­ecular structure with displacement ellipsoids drawn at the 50% probability level and H atoms are omitted for clarity.

**Fig. 2.**
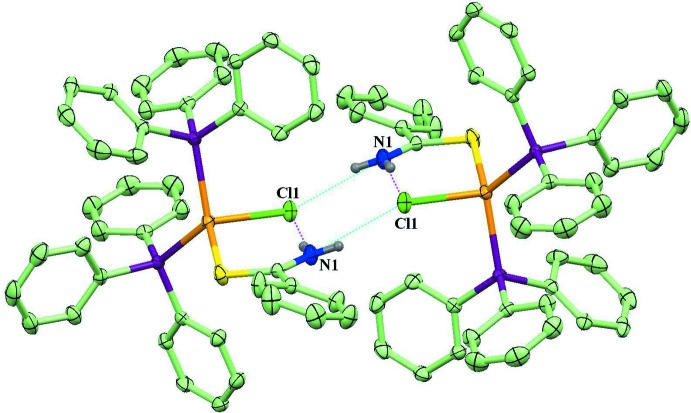
Part of the crystal structure showing the intermolecular and intramolecular hydrogen bonds as dashed lines are drawn between the non-hydrogen atoms. The symmetry related molecule is generated by the operator (-x+2, -y, -z).

. DOI: 10.1107/S1600536814015992/lh5718fig2.tif
Part of the crystal structure showing the inter­molecular and intra­molecular hydrogen bonds as dashed lines are drawn between the non-hydrogen atoms. The symmetry related mol­ecule is generated by the operator (-x+2, −y, −z).

CCDC reference: 1012936

Additional supporting information:  crystallographic information; 3D view; checkCIF report

## Figures and Tables

**Table 1:** Hydrogen-bond geometry (Å, °)

*D*—H⋯*A*	*D*—H	H⋯*A*	*D*⋯*A*	*D*—H⋯*A*
N1—H1*A*⋯Cl1^i^	0.86	2.43	3.1798 (17)	146
N1—H1*B*⋯Cl1	0.86	2.39	3.2434 (17)	173

Symmetry code: (i) 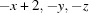.

## supplementary crystallographic information

### S1. Experimental

Tri­phenyl­phosphine, (0.37 g, 0.14 mmol) was dissolved in 30 cm^3^ of aceto­nitrile at 343 K. AgCl (0.10 g, 0.70 mmol) was added and then the mixture was stirred for 3 hours. At that time, a white precipitate was deposited and then benzene­carbo­thio­amide (0.09 g, 0.70 mmol) was added. The new reaction mixture was continually heated under refluxed for 5 hours. The resulting clear solution was filtered and the filtrate was kept to evaporate at room temperature. X-ray quality crystals were obtained after two days and were filtered and dried in *vacuo*.

### S1.1. Refinement

All H atoms were positioned geometrically and refined using a riding-model, approximation with C—H = 0.93 with *U_iso_*(H) = 1.2 *U_eq_*(C), N—H = 0.86 with *U_iso_*(H) = 1.2 *U_eq_*(N).

### S2. Results and discussion

The coordination compounds of Ag^I^ complexes with phosphine and thione ligands have gained inter­est in recent years because of their participation in biological process as anti­bacterial agents (Isab *et al.*, 2010; Nawaz *et al.*, 2011). A large number of crystal structures of Ag^I^ complexes containing phosphine and thione as ligands have presented that these complexes are either mononuclear (McFarlane *et al.*, 1998) or dinuclear (Cox *et al*., 2000) complexes with the silver(I) center exhibiting distorted tetra­hedral or trigonal coordination geometry (Aslanidis *et al.*, 1997).

The molecular structure of the title mononuclear complex displays a distorted tetra­hedral coordination geometry (Fig. 1). The deviation from ideal tetra­hedral geometry can be described by the P2—Ag1—P1 angle which has value of 120.05 (2)° and this value is much larger than the perfect tetra­hedral value of 109.5°. This distortion may be due to the steric imposition induced by the two bulky tri­phenyl­phosphine ligands. This has also been observed in previous reports e.g. the P2—Cu1—P1 angle in [CuI(C_7_H_8_N_2_S)(C_18_H_15_P)_2_] is 118.63 (5)° (Nimthong *et al.*, 2008). The Ag1—S1 bond distance in the title complex 2.5880 (5) Å lies in the range of other Ag^I^ complexes such as the Ag—S bond in [Ag(C_7_H_4_NS_2_O_2_)(C_18_H_15_P)_3_] of 2.5939 (10) Å (Dennehy *et al*., 2007). In the crystal, pairs of N—H···Cl hydrogen bonds form inversion dimers. An intra­molecular N—H···Cl hydrogen bond is also observed (Fig. 2).

### Crystal data

**Table:**

[AgCl(C_7_H_7_NS)(C_18_H_15_P)_2_]	*F*(000) = 1648
*M**_r_* = 805.05	*D*_x_ = 1.430 Mg m^−^^3^
Monoclinic, *P*2_1_/*n*	Mo *K*α radiation, λ = 0.71073 Å
*a* = 14.2405 (7) Å	Cell parameters from 7876 reflections
*b* = 13.9271 (7) Å	θ = 2.4–28.3°
*c* = 19.0774 (10) Å	µ = 0.78 mm^−^^1^
β = 98.711 (1)°	*T* = 293 K
*V* = 3740.0 (3) Å^3^	Block, colorless
*Z* = 4	0.30 × 0.16 × 0.10 mm

### Data collection

**Table:**

Bruker SMART APEX CCD diffractometer	8544 reflections with *I* > 2σ(*I*)
Radiation source: sealed tube	*R*_int_ = 0.031
φ and ω scans	θ_max_ = 28.3°, θ_min_ = 1.7°
Absorption correction: multi-scan (*SADABS*; Sheldrick, 2003)	*h* = −19→18
*T*_min_ = 0.861, *T*_max_ = 0.923	*k* = −18→18
51284 measured reflections	*l* = −25→25
9308 independent reflections	

### Refinement

**Table:**

Refinement on *F*^2^	0 restraints
Least-squares matrix: full	Hydrogen site location: inferred from neighbouring sites
*R*[*F*^2^ > 2σ(*F*^2^)] = 0.033	H-atom parameters constrained
*wR*(*F*^2^) = 0.075	*w* = 1/[σ^2^(*F*_o_^2^) + (0.0351*P*)^2^ + 1.9067*P*] where *P* = (*F*_o_^2^ + 2*F*_c_^2^)/3
*S* = 1.13	(Δ/σ)_max_ = 0.001
9308 reflections	Δρ_max_ = 0.72 e Å^−^^3^
442 parameters	Δρ_min_ = −0.39 e Å^−^^3^

### Special details

**Table:**

Geometry. All e.s.d.'s (except the e.s.d. in the dihedral angle between two l.s. planes) are estimated using the full covariance matrix. The cell e.s.d.'s are taken into account individually in the estimation of e.s.d.'s in distances, angles and torsion angles; correlations between e.s.d.'s in cell parameters are only used when they are defined by crystal symmetry. An approximate (isotropic) treatment of cell e.s.d.'s is used for estimating e.s.d.'s involving l.s. planes.

### Fractional atomic coordinates and isotropic or equivalent isotropic displacement parameters (Å^2^)

**Table:**

	*x*	*y*	*z*	*U*_iso_*/*U*_eq_	
Ag1	0.96337 (2)	0.11780 (2)	0.18826 (2)	0.01933 (5)	
Cl1	1.07616 (3)	0.00745 (4)	0.12713 (2)	0.02523 (10)	
S1	0.79118 (3)	0.07930 (4)	0.13044 (2)	0.02617 (11)	
P1	1.02572 (3)	0.27274 (3)	0.15422 (2)	0.01705 (9)	
P2	0.98356 (3)	0.07020 (3)	0.31343 (2)	0.01641 (9)	
N1	0.86973 (12)	−0.00117 (13)	0.02851 (9)	0.0257 (4)	
H1A	0.8678	−0.0242	−0.0135	0.031*	
H1B	0.9221	−0.0018	0.0576	0.031*	
C1	1.02873 (12)	0.29144 (13)	0.05997 (9)	0.0187 (3)	
C2	1.05820 (14)	0.37795 (14)	0.03329 (10)	0.0236 (4)	
H2	1.0772	0.4285	0.0640	0.028*	
C3	1.05912 (15)	0.38854 (15)	−0.03897 (11)	0.0268 (4)	
H3	1.0791	0.4460	−0.0566	0.032*	
C4	1.03040 (16)	0.31370 (16)	−0.08444 (11)	0.0296 (4)	
H4	1.0312	0.3210	−0.1328	0.036*	
C5	1.00051 (17)	0.22818 (16)	−0.05892 (11)	0.0324 (5)	
H5	0.9810	0.1781	−0.0899	0.039*	
C6	0.99972 (15)	0.21714 (14)	0.01362 (10)	0.0258 (4)	
H6	0.9796	0.1595	0.0309	0.031*	
C7	1.14768 (13)	0.29086 (13)	0.19631 (10)	0.0200 (4)	
C8	1.16664 (14)	0.30735 (15)	0.26924 (10)	0.0263 (4)	
H8	1.1166	0.3123	0.2952	0.032*	
C9	1.25915 (16)	0.31638 (17)	0.30335 (12)	0.0352 (5)	
H9	1.2711	0.3289	0.3518	0.042*	
C10	1.33373 (16)	0.30669 (18)	0.26532 (13)	0.0374 (5)	
H10	1.3960	0.3129	0.2881	0.045*	
C11	1.31608 (15)	0.28778 (18)	0.19375 (13)	0.0377 (5)	
H11	1.3665	0.2800	0.1685	0.045*	
C12	1.22346 (14)	0.28022 (16)	0.15898 (11)	0.0286 (4)	
H12	1.2121	0.2680	0.1105	0.034*	
C13	0.96006 (13)	0.37748 (13)	0.17718 (10)	0.0197 (4)	
C14	0.86150 (15)	0.37148 (15)	0.16127 (13)	0.0305 (5)	
H14	0.8335	0.3157	0.1411	0.037*	
C15	0.80496 (16)	0.44786 (18)	0.17519 (14)	0.0390 (6)	
H15	0.7393	0.4438	0.1632	0.047*	
C16	0.84564 (17)	0.53014 (17)	0.20690 (12)	0.0348 (5)	
H16	0.8075	0.5811	0.2166	0.042*	
C17	0.94290 (16)	0.53631 (15)	0.22403 (10)	0.0286 (4)	
H17	0.9702	0.5913	0.2460	0.034*	
C18	1.00066 (14)	0.46105 (14)	0.20872 (10)	0.0223 (4)	
H18	1.0664	0.4664	0.2195	0.027*	
C19	1.10059 (13)	0.08806 (13)	0.36558 (10)	0.0193 (4)	
C20	1.11456 (15)	0.12565 (17)	0.43381 (11)	0.0319 (5)	
H20	1.0625	0.1439	0.4548	0.038*	
C21	1.20579 (16)	0.13612 (19)	0.47091 (13)	0.0386 (5)	
H21	1.2144	0.1611	0.5167	0.046*	
C22	1.28319 (15)	0.10983 (17)	0.44038 (13)	0.0358 (5)	
H22	1.3442	0.1164	0.4654	0.043*	
C23	1.26999 (16)	0.07346 (19)	0.37215 (14)	0.0385 (5)	
H23	1.3224	0.0563	0.3511	0.046*	
C24	1.17911 (14)	0.06228 (16)	0.33477 (12)	0.0297 (4)	
H24	1.1709	0.0375	0.2890	0.036*	
C25	0.90286 (13)	0.13427 (14)	0.36321 (10)	0.0201 (4)	
C26	0.86802 (14)	0.09695 (16)	0.42206 (11)	0.0272 (4)	
H26	0.8856	0.0355	0.4380	0.033*	
C27	0.80725 (15)	0.15072 (19)	0.45705 (12)	0.0362 (5)	
H27	0.7838	0.1249	0.4959	0.043*	
C28	0.78167 (16)	0.2422 (2)	0.43432 (13)	0.0413 (6)	
H28	0.7413	0.2781	0.4580	0.050*	
C29	0.81590 (16)	0.28085 (17)	0.37632 (14)	0.0381 (5)	
H29	0.7990	0.3429	0.3613	0.046*	
C30	0.87564 (14)	0.22677 (15)	0.34051 (11)	0.0280 (4)	
H30	0.8977	0.2525	0.3011	0.034*	
C31	0.96144 (13)	−0.05724 (13)	0.32671 (9)	0.0185 (3)	
C32	0.91259 (14)	−0.10887 (14)	0.26999 (11)	0.0246 (4)	
H32	0.8938	−0.0785	0.2267	0.030*	
C33	0.89178 (15)	−0.20533 (15)	0.27774 (12)	0.0297 (4)	
H33	0.8587	−0.2391	0.2398	0.036*	
C34	0.92006 (16)	−0.25143 (15)	0.34174 (12)	0.0295 (4)	
H34	0.9052	−0.3158	0.3471	0.035*	
C35	0.97056 (16)	−0.20123 (15)	0.39764 (11)	0.0288 (4)	
H35	0.9907	−0.2323	0.4404	0.035*	
C36	0.99138 (14)	−0.10483 (14)	0.39031 (10)	0.0243 (4)	
H36	1.0256	−0.0717	0.4282	0.029*	
C37	0.79299 (13)	0.03483 (13)	0.04814 (9)	0.0200 (4)	
C38	0.70443 (14)	0.03396 (14)	−0.00449 (9)	0.0219 (4)	
C39	0.69078 (16)	−0.03464 (16)	−0.05811 (11)	0.0307 (5)	
H39	0.7379	−0.0797	−0.0620	0.037*	
C40	0.60665 (17)	−0.03594 (18)	−0.10586 (12)	0.0379 (5)	
H40	0.5977	−0.0820	−0.1415	0.045*	
C41	0.53653 (16)	0.03057 (19)	−0.10061 (12)	0.0386 (5)	
H41	0.4800	0.0289	−0.1323	0.046*	
C42	0.55038 (18)	0.0996 (2)	−0.04827 (13)	0.0444 (6)	
H42	0.5035	0.1451	−0.0450	0.053*	
C43	0.63401 (16)	0.10130 (18)	−0.00047 (12)	0.0348 (5)	
H43	0.6429	0.1482	0.0346	0.042*	

### Atomic displacement parameters (Å^2^)

**Table:**

	*U*^11^	*U*^22^	*U*^33^	*U*^12^	*U*^13^	*U*^23^
Ag1	0.02407 (8)	0.01923 (7)	0.01454 (7)	−0.00135 (5)	0.00242 (5)	0.00012 (5)
Cl1	0.0264 (2)	0.0283 (2)	0.0204 (2)	0.00856 (19)	0.00159 (17)	−0.00369 (18)
S1	0.0203 (2)	0.0411 (3)	0.0165 (2)	0.0002 (2)	0.00093 (17)	−0.0062 (2)
P1	0.0174 (2)	0.0171 (2)	0.0162 (2)	−0.00025 (17)	0.00140 (16)	0.00061 (16)
P2	0.0171 (2)	0.0186 (2)	0.0133 (2)	−0.00141 (17)	0.00131 (16)	−0.00025 (16)
N1	0.0225 (8)	0.0345 (9)	0.0191 (8)	0.0021 (7)	−0.0006 (6)	−0.0072 (7)
C1	0.0167 (8)	0.0210 (9)	0.0179 (8)	0.0013 (7)	0.0012 (6)	0.0017 (7)
C2	0.0261 (10)	0.0208 (9)	0.0237 (9)	−0.0039 (7)	0.0035 (7)	−0.0005 (7)
C3	0.0301 (10)	0.0256 (10)	0.0261 (10)	−0.0010 (8)	0.0083 (8)	0.0060 (8)
C4	0.0378 (12)	0.0331 (11)	0.0185 (9)	0.0057 (9)	0.0058 (8)	0.0053 (8)
C5	0.0494 (14)	0.0257 (10)	0.0201 (9)	−0.0008 (9)	−0.0013 (9)	−0.0028 (8)
C6	0.0353 (11)	0.0210 (9)	0.0199 (9)	−0.0015 (8)	0.0000 (8)	0.0034 (7)
C7	0.0192 (8)	0.0178 (8)	0.0220 (9)	0.0003 (7)	0.0000 (7)	0.0005 (7)
C8	0.0250 (10)	0.0304 (10)	0.0226 (9)	0.0018 (8)	0.0007 (8)	−0.0007 (8)
C9	0.0344 (12)	0.0376 (12)	0.0295 (11)	0.0001 (10)	−0.0083 (9)	−0.0021 (9)
C10	0.0222 (10)	0.0395 (13)	0.0463 (13)	−0.0031 (9)	−0.0088 (9)	0.0019 (10)
C11	0.0198 (10)	0.0463 (14)	0.0478 (14)	0.0014 (9)	0.0072 (9)	0.0036 (11)
C12	0.0241 (10)	0.0350 (11)	0.0265 (10)	0.0017 (8)	0.0036 (8)	−0.0010 (8)
C13	0.0207 (9)	0.0209 (9)	0.0183 (8)	0.0029 (7)	0.0052 (7)	0.0039 (7)
C14	0.0216 (10)	0.0261 (10)	0.0439 (13)	0.0003 (8)	0.0050 (9)	0.0054 (9)
C15	0.0228 (10)	0.0388 (13)	0.0580 (15)	0.0107 (9)	0.0139 (10)	0.0135 (11)
C16	0.0421 (13)	0.0315 (11)	0.0346 (11)	0.0170 (10)	0.0180 (10)	0.0103 (9)
C17	0.0449 (12)	0.0240 (10)	0.0170 (9)	0.0079 (9)	0.0050 (8)	0.0014 (7)
C18	0.0265 (9)	0.0214 (9)	0.0187 (8)	0.0022 (7)	0.0019 (7)	0.0017 (7)
C19	0.0175 (8)	0.0193 (8)	0.0200 (8)	−0.0007 (7)	−0.0007 (7)	0.0022 (7)
C20	0.0225 (10)	0.0473 (13)	0.0255 (10)	−0.0013 (9)	0.0019 (8)	−0.0068 (9)
C21	0.0291 (11)	0.0536 (15)	0.0298 (11)	−0.0051 (10)	−0.0064 (9)	−0.0102 (10)
C22	0.0199 (10)	0.0411 (13)	0.0429 (13)	−0.0012 (9)	−0.0070 (9)	0.0014 (10)
C23	0.0201 (10)	0.0462 (14)	0.0494 (14)	0.0062 (9)	0.0065 (9)	−0.0019 (11)
C24	0.0239 (10)	0.0342 (11)	0.0308 (11)	0.0024 (8)	0.0037 (8)	−0.0061 (9)
C25	0.0151 (8)	0.0247 (9)	0.0196 (8)	−0.0004 (7)	−0.0001 (7)	−0.0053 (7)
C26	0.0212 (9)	0.0377 (12)	0.0229 (9)	−0.0005 (8)	0.0041 (7)	−0.0030 (8)
C27	0.0250 (11)	0.0556 (15)	0.0297 (11)	−0.0022 (10)	0.0096 (9)	−0.0119 (10)
C28	0.0259 (11)	0.0541 (16)	0.0441 (14)	0.0043 (10)	0.0062 (10)	−0.0252 (12)
C29	0.0311 (11)	0.0312 (12)	0.0499 (14)	0.0070 (9)	−0.0004 (10)	−0.0121 (10)
C30	0.0256 (10)	0.0264 (10)	0.0307 (10)	0.0014 (8)	0.0001 (8)	−0.0041 (8)
C31	0.0173 (8)	0.0197 (9)	0.0194 (8)	−0.0008 (7)	0.0060 (7)	−0.0005 (7)
C32	0.0263 (10)	0.0244 (10)	0.0220 (9)	−0.0008 (8)	0.0003 (8)	−0.0021 (7)
C33	0.0325 (11)	0.0233 (10)	0.0327 (11)	−0.0047 (8)	0.0029 (9)	−0.0080 (8)
C34	0.0345 (11)	0.0193 (9)	0.0384 (11)	−0.0029 (8)	0.0170 (9)	−0.0016 (8)
C35	0.0393 (12)	0.0250 (10)	0.0245 (10)	0.0002 (9)	0.0123 (9)	0.0053 (8)
C36	0.0292 (10)	0.0254 (10)	0.0186 (9)	−0.0019 (8)	0.0049 (8)	−0.0005 (7)
C37	0.0227 (9)	0.0198 (9)	0.0172 (8)	−0.0031 (7)	0.0017 (7)	0.0003 (7)
C38	0.0231 (9)	0.0255 (9)	0.0162 (8)	−0.0035 (7)	−0.0003 (7)	0.0003 (7)
C39	0.0307 (11)	0.0296 (11)	0.0292 (10)	0.0013 (9)	−0.0034 (8)	−0.0070 (9)
C40	0.0370 (12)	0.0416 (13)	0.0309 (11)	−0.0022 (10)	−0.0080 (9)	−0.0118 (10)
C41	0.0292 (11)	0.0586 (16)	0.0242 (10)	0.0023 (11)	−0.0082 (9)	−0.0049 (10)
C42	0.0349 (13)	0.0619 (17)	0.0326 (12)	0.0199 (12)	−0.0072 (10)	−0.0099 (11)
C43	0.0336 (12)	0.0441 (13)	0.0239 (10)	0.0105 (10)	−0.0042 (9)	−0.0115 (9)

### Geometric parameters (Å, º)

**Table:**

Ag1—P2	2.4529 (5)	C18—H18	0.9300
Ag1—P1	2.4578 (5)	C19—C24	1.387 (3)
Ag1—S1	2.5880 (5)	C19—C20	1.389 (3)
Ag1—Cl1	2.6208 (5)	C20—C21	1.390 (3)
S1—C37	1.6918 (19)	C20—H20	0.9300
P1—C7	1.8173 (19)	C21—C22	1.372 (3)
P1—C13	1.8216 (19)	C21—H21	0.9300
P1—C1	1.8238 (18)	C22—C23	1.383 (3)
P2—C19	1.8240 (18)	C22—H22	0.9300
P2—C31	1.8269 (19)	C23—C24	1.389 (3)
P2—C25	1.8300 (19)	C23—H23	0.9300
N1—C37	1.308 (2)	C24—H24	0.9300
N1—H1A	0.8600	C25—C26	1.395 (3)
N1—H1B	0.8600	C25—C30	1.395 (3)
C1—C6	1.383 (3)	C26—C27	1.389 (3)
C1—C2	1.397 (3)	C26—H26	0.9300
C2—C3	1.388 (3)	C27—C28	1.377 (4)
C2—H2	0.9300	C27—H27	0.9300
C3—C4	1.378 (3)	C28—C29	1.384 (4)
C3—H3	0.9300	C28—H28	0.9300
C4—C5	1.378 (3)	C29—C30	1.391 (3)
C4—H4	0.9300	C29—H29	0.9300
C5—C6	1.394 (3)	C30—H30	0.9300
C5—H5	0.9300	C31—C36	1.392 (3)
C6—H6	0.9300	C31—C32	1.394 (3)
C7—C12	1.388 (3)	C32—C33	1.388 (3)
C7—C8	1.396 (3)	C32—H32	0.9300
C8—C9	1.384 (3)	C33—C34	1.384 (3)
C8—H8	0.9300	C33—H33	0.9300
C9—C10	1.381 (3)	C34—C35	1.382 (3)
C9—H9	0.9300	C34—H34	0.9300
C10—C11	1.376 (3)	C35—C36	1.386 (3)
C10—H10	0.9300	C35—H35	0.9300
C11—C12	1.388 (3)	C36—H36	0.9300
C11—H11	0.9300	C37—C38	1.488 (2)
C12—H12	0.9300	C38—C43	1.384 (3)
C13—C14	1.393 (3)	C38—C39	1.392 (3)
C13—C18	1.395 (3)	C39—C40	1.391 (3)
C14—C15	1.384 (3)	C39—H39	0.9300
C14—H14	0.9300	C40—C41	1.377 (3)
C15—C16	1.381 (4)	C40—H40	0.9300
C15—H15	0.9300	C41—C42	1.378 (3)
C16—C17	1.377 (3)	C41—H41	0.9300
C16—H16	0.9300	C42—C43	1.386 (3)
C17—C18	1.390 (3)	C42—H42	0.9300
C17—H17	0.9300	C43—H43	0.9300
			
P2—Ag1—P1	120.053 (16)	C24—C19—C20	118.98 (18)
P2—Ag1—S1	108.823 (16)	C24—C19—P2	117.53 (15)
P1—Ag1—S1	115.223 (17)	C20—C19—P2	123.49 (15)
P2—Ag1—Cl1	106.579 (16)	C19—C20—C21	120.5 (2)
P1—Ag1—Cl1	97.298 (16)	C19—C20—H20	119.8
S1—Ag1—Cl1	107.087 (16)	C21—C20—H20	119.8
C37—S1—Ag1	108.95 (7)	C22—C21—C20	120.3 (2)
C7—P1—C13	105.87 (9)	C22—C21—H21	119.8
C7—P1—C1	104.61 (8)	C20—C21—H21	119.8
C13—P1—C1	102.26 (8)	C21—C22—C23	119.6 (2)
C7—P1—Ag1	111.37 (6)	C21—C22—H22	120.2
C13—P1—Ag1	114.86 (6)	C23—C22—H22	120.2
C1—P1—Ag1	116.70 (6)	C22—C23—C24	120.5 (2)
C19—P2—C31	102.67 (8)	C22—C23—H23	119.7
C19—P2—C25	104.13 (8)	C24—C23—H23	119.7
C31—P2—C25	105.47 (9)	C19—C24—C23	120.1 (2)
C19—P2—Ag1	117.18 (6)	C19—C24—H24	119.9
C31—P2—Ag1	113.60 (6)	C23—C24—H24	119.9
C25—P2—Ag1	112.53 (6)	C26—C25—C30	118.54 (18)
C37—N1—H1A	120.0	C26—C25—P2	124.32 (15)
C37—N1—H1B	120.0	C30—C25—P2	117.13 (15)
H1A—N1—H1B	120.0	C27—C26—C25	120.6 (2)
C6—C1—C2	119.31 (17)	C27—C26—H26	119.7
C6—C1—P1	118.31 (14)	C25—C26—H26	119.7
C2—C1—P1	122.38 (14)	C28—C27—C26	120.1 (2)
C3—C2—C1	120.12 (18)	C28—C27—H27	119.9
C3—C2—H2	119.9	C26—C27—H27	119.9
C1—C2—H2	119.9	C27—C28—C29	120.2 (2)
C4—C3—C2	119.88 (18)	C27—C28—H28	119.9
C4—C3—H3	120.1	C29—C28—H28	119.9
C2—C3—H3	120.1	C28—C29—C30	119.9 (2)
C3—C4—C5	120.62 (19)	C28—C29—H29	120.1
C3—C4—H4	119.7	C30—C29—H29	120.1
C5—C4—H4	119.7	C29—C30—C25	120.6 (2)
C4—C5—C6	119.69 (19)	C29—C30—H30	119.7
C4—C5—H5	120.2	C25—C30—H30	119.7
C6—C5—H5	120.2	C36—C31—C32	118.82 (17)
C1—C6—C5	120.38 (19)	C36—C31—P2	123.17 (14)
C1—C6—H6	119.8	C32—C31—P2	118.01 (14)
C5—C6—H6	119.8	C33—C32—C31	120.38 (19)
C12—C7—C8	118.76 (18)	C33—C32—H32	119.8
C12—C7—P1	121.58 (15)	C31—C32—H32	119.8
C8—C7—P1	119.41 (15)	C34—C33—C32	120.30 (19)
C9—C8—C7	120.7 (2)	C34—C33—H33	119.9
C9—C8—H8	119.7	C32—C33—H33	119.9
C7—C8—H8	119.7	C35—C34—C33	119.59 (19)
C10—C9—C8	119.8 (2)	C35—C34—H34	120.2
C10—C9—H9	120.1	C33—C34—H34	120.2
C8—C9—H9	120.1	C34—C35—C36	120.40 (19)
C11—C10—C9	120.0 (2)	C34—C35—H35	119.8
C11—C10—H10	120.0	C36—C35—H35	119.8
C9—C10—H10	120.0	C35—C36—C31	120.48 (19)
C10—C11—C12	120.4 (2)	C35—C36—H36	119.8
C10—C11—H11	119.8	C31—C36—H36	119.8
C12—C11—H11	119.8	N1—C37—C38	117.60 (16)
C11—C12—C7	120.2 (2)	N1—C37—S1	122.48 (14)
C11—C12—H12	119.9	C38—C37—S1	119.91 (14)
C7—C12—H12	119.9	C43—C38—C39	118.97 (18)
C14—C13—C18	118.83 (18)	C43—C38—C37	120.29 (17)
C14—C13—P1	115.88 (15)	C39—C38—C37	120.74 (18)
C18—C13—P1	125.29 (14)	C40—C39—C38	120.1 (2)
C15—C14—C13	120.6 (2)	C40—C39—H39	120.0
C15—C14—H14	119.7	C38—C39—H39	120.0
C13—C14—H14	119.7	C41—C40—C39	120.4 (2)
C16—C15—C14	120.3 (2)	C41—C40—H40	119.8
C16—C15—H15	119.9	C39—C40—H40	119.8
C14—C15—H15	119.9	C40—C41—C42	119.7 (2)
C17—C16—C15	119.7 (2)	C40—C41—H41	120.1
C17—C16—H16	120.1	C42—C41—H41	120.1
C15—C16—H16	120.1	C41—C42—C43	120.2 (2)
C16—C17—C18	120.6 (2)	C41—C42—H42	119.9
C16—C17—H17	119.7	C43—C42—H42	119.9
C18—C17—H17	119.7	C38—C43—C42	120.6 (2)
C17—C18—C13	119.99 (19)	C38—C43—H43	119.7
C17—C18—H18	120.0	C42—C43—H43	119.7
C13—C18—H18	120.0		
			
C7—P1—C1—C6	121.08 (16)	P2—C19—C20—C21	179.13 (18)
C13—P1—C1—C6	−128.69 (16)	C19—C20—C21—C22	0.3 (4)
Ag1—P1—C1—C6	−2.47 (17)	C20—C21—C22—C23	0.5 (4)
C7—P1—C1—C2	−59.59 (17)	C21—C22—C23—C24	−0.8 (4)
C13—P1—C1—C2	50.64 (17)	C20—C19—C24—C23	0.5 (3)
Ag1—P1—C1—C2	176.86 (13)	P2—C19—C24—C23	−179.44 (18)
C6—C1—C2—C3	−0.6 (3)	C22—C23—C24—C19	0.3 (4)
P1—C1—C2—C3	−179.96 (15)	C19—P2—C25—C26	−81.47 (18)
C1—C2—C3—C4	0.4 (3)	C31—P2—C25—C26	26.25 (18)
C2—C3—C4—C5	0.1 (3)	Ag1—P2—C25—C26	150.62 (15)
C3—C4—C5—C6	−0.3 (3)	C19—P2—C25—C30	97.99 (15)
C2—C1—C6—C5	0.5 (3)	C31—P2—C25—C30	−154.29 (14)
P1—C1—C6—C5	179.81 (16)	Ag1—P2—C25—C30	−29.92 (16)
C4—C5—C6—C1	0.0 (3)	C30—C25—C26—C27	0.2 (3)
C13—P1—C7—C12	−130.56 (17)	P2—C25—C26—C27	179.70 (16)
C1—P1—C7—C12	−22.97 (19)	C25—C26—C27—C28	−0.8 (3)
Ag1—P1—C7—C12	103.94 (16)	C26—C27—C28—C29	0.4 (3)
C13—P1—C7—C8	55.30 (17)	C27—C28—C29—C30	0.6 (3)
C1—P1—C7—C8	162.89 (16)	C28—C29—C30—C25	−1.1 (3)
Ag1—P1—C7—C8	−70.20 (16)	C26—C25—C30—C29	0.7 (3)
C12—C7—C8—C9	2.3 (3)	P2—C25—C30—C29	−178.80 (16)
P1—C7—C8—C9	176.59 (17)	C19—P2—C31—C36	35.53 (18)
C7—C8—C9—C10	−1.6 (3)	C25—P2—C31—C36	−73.24 (17)
C8—C9—C10—C11	−0.2 (4)	Ag1—P2—C31—C36	163.06 (14)
C9—C10—C11—C12	1.3 (4)	C19—P2—C31—C32	−144.12 (15)
C10—C11—C12—C7	−0.6 (4)	C25—P2—C31—C32	107.10 (16)
C8—C7—C12—C11	−1.2 (3)	Ag1—P2—C31—C32	−16.59 (17)
P1—C7—C12—C11	−175.38 (17)	C36—C31—C32—C33	2.0 (3)
C7—P1—C13—C14	−168.93 (15)	P2—C31—C32—C33	−178.37 (16)
C1—P1—C13—C14	81.80 (16)	C31—C32—C33—C34	−0.5 (3)
Ag1—P1—C13—C14	−45.60 (17)	C32—C33—C34—C35	−1.1 (3)
C7—P1—C13—C18	11.10 (18)	C33—C34—C35—C36	1.2 (3)
C1—P1—C13—C18	−98.18 (17)	C34—C35—C36—C31	0.2 (3)
Ag1—P1—C13—C18	134.42 (15)	C32—C31—C36—C35	−1.8 (3)
C18—C13—C14—C15	1.3 (3)	P2—C31—C36—C35	178.54 (15)
P1—C13—C14—C15	−178.66 (18)	Ag1—S1—C37—N1	19.52 (18)
C13—C14—C15—C16	−1.8 (4)	Ag1—S1—C37—C38	−161.76 (13)
C14—C15—C16—C17	0.6 (3)	N1—C37—C38—C43	−153.7 (2)
C15—C16—C17—C18	1.0 (3)	S1—C37—C38—C43	27.5 (3)
C16—C17—C18—C13	−1.5 (3)	N1—C37—C38—C39	26.7 (3)
C14—C13—C18—C17	0.3 (3)	S1—C37—C38—C39	−152.13 (17)
P1—C13—C18—C17	−179.73 (14)	C43—C38—C39—C40	−1.1 (3)
C31—P2—C19—C24	81.55 (17)	C37—C38—C39—C40	178.5 (2)
C25—P2—C19—C24	−168.66 (16)	C38—C39—C40—C41	0.2 (4)
Ag1—P2—C19—C24	−43.67 (18)	C39—C40—C41—C42	0.8 (4)
C31—P2—C19—C20	−98.36 (18)	C40—C41—C42—C43	−0.9 (4)
C25—P2—C19—C20	11.4 (2)	C39—C38—C43—C42	1.1 (4)
Ag1—P2—C19—C20	136.42 (16)	C37—C38—C43—C42	−178.5 (2)
C24—C19—C20—C21	−0.8 (3)	C41—C42—C43—C38	−0.1 (4)

### Hydrogen-bond geometry (Å, º)

**Table:**

*D*—H···*A*	*D*—H	H···*A*	*D*···*A*	*D*—H···*A*
N1—H1*A*···Cl1^i^	0.86	2.43	3.1798 (17)	146
N1—H1*B*···Cl1	0.86	2.39	3.2434 (17)	173

Symmetry code: (i) −*x*+2, −*y*, −*z*.
