# Supplementary figures and images for: Crystal structure of (benzene­carbo­thio­amide-κS)chloridobis­(tri­phenyl­phosphane-κP)silver(I)
Source: Acta Crystallogr Sect E Struct Rep Online. 2014 Aug 1;70(Pt 9):m310–1. doi: 10.1107/S1600536814015992 (PMC4186092; doi:10.1107/S1600536814015992)

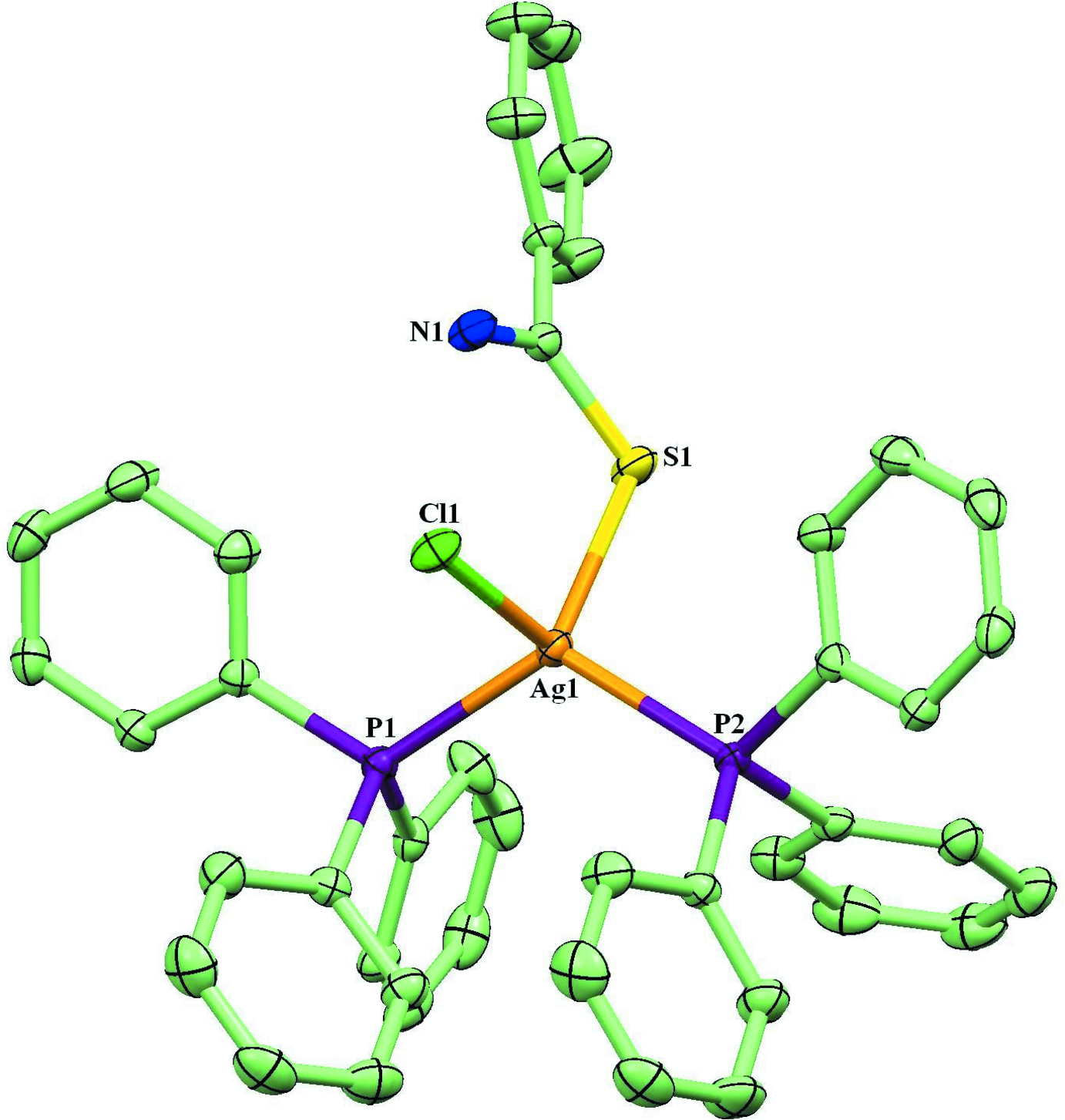

Supplement: Supplementary file 3 [file e-70-0m310-fig1.tif]

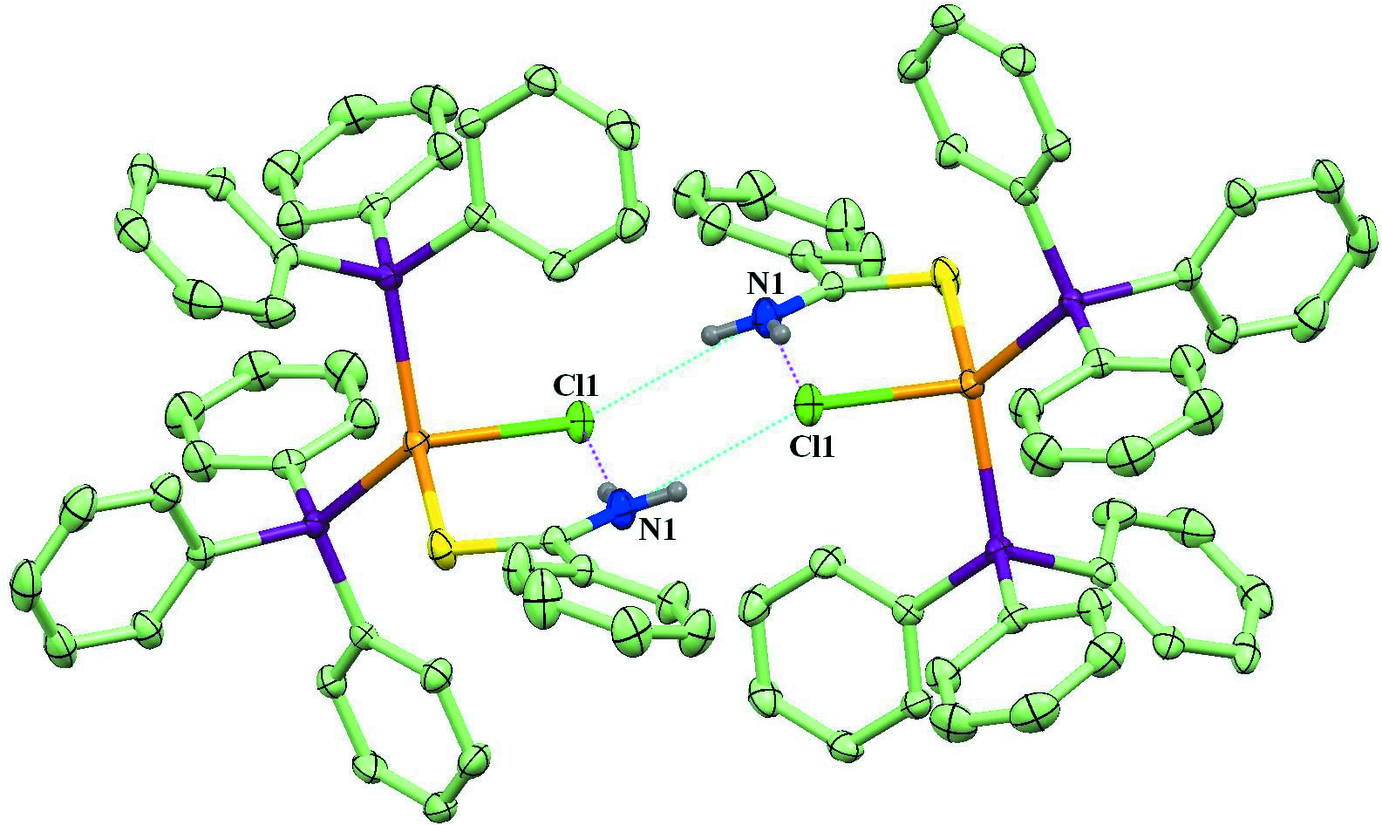

Supplement: Supplementary file 4 [file e-70-0m310-fig2.tif]
